# The Class A Carbapenemases BKC-1 and GPC-1 Both Originate from the Bacterial Genus *Shinella*

**DOI:** 10.1128/AAC.01263-20

**Published:** 2020-11-17

**Authors:** Nicolas Kieffer, Stefan Ebmeyer, D. G. Joakim Larsson

**Affiliations:** a Centre for Antibiotic Resistance Research (CARe), University of Gothenburg, Gothenburg, Sweden; b Department of Infectious Diseases, Institute of Biomedicine, Sahlgrenska Academy, University of Gothenburg, Gothenburg, Sweden

**Keywords:** class A, β-lactamase, carbapenemase, origin, environment, Gram-negative bacteria, antimicrobial resistance, antibiotic resistance

## Abstract

Comparative genomics identified the environmental bacterial genus *Shinella* as the most likely origin of the class A carbapenemases BKC-1 and GPC-1. Available sequences and PCR analyses of additional *Shinella* species revealed homologous β-lactamases showing up to 85.4% and 93.3% amino acid identity to both enzymes, respectively. The genes conferred resistance to β-lactams once expressed in Escherichia coli. *bla*_BKC-1_ likely evolved from a putative ancestral *Shinella* gene with higher homology through duplication of a gene fragment.

## INTRODUCTION

The high potential of Gram-negative bacteria to acquire exogenous DNA through horizontal gene transfer has allowed clinically relevant bacteria to acquire resistance toward many antibiotics (1, 2). The acquisition of carbapenemase genes in *Enterobacteriaceae*, Pseudomonas aeruginosa, and Acinetobacter baumannii represents one of the most important threats, compromising the use of the entire β-lactam family.

Recently, two novel carbapenemase genes, *bla*_BKC-1_ and *bla*_GPC-1_, were characterized (3, 4). The genes code for weak class A carbapenemases sporadically identified in Klebsiella pneumoniae and P. aeruginosa isolates, respectively. The two enzymes, BKC-1 and GPC-1, share 77% amino acid identity, but their exact origins remain unknown. The aim of this study was to investigate the origin of both *bla*_BKC-1_ and *bla*_GPC-1_.

**FIG 1:**
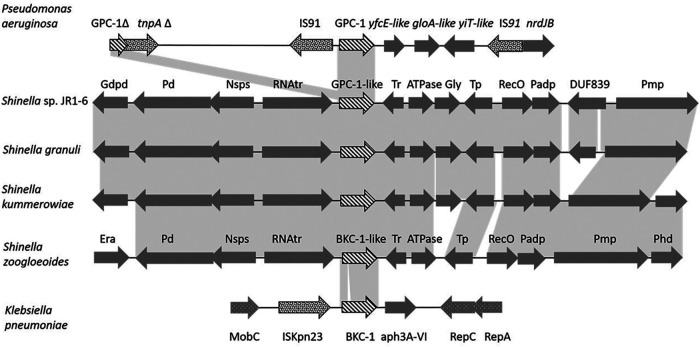
Comparative analysis of GPC-1/BKC-1-like loci. Striped arrows denote GPC-1/BKC-1-like genes, dark spotted arrows symbolize transposition associated genes such as IS, and light spotted arrows denote other genes associated with mobility. Light gray areas between graphs symbolize sequence alignment. Nucleotide alignment identities between GPC-1/BKC-1-like loci top to bottom: P. aeruginosa to *S*. spp JR1-6: 89% to 90%; *S*. spp JR1-6 to *S. granuli*, 70% to 93%; *S. granuli* to *S. kummerowiae*, 84% to 88%; *S. kummerowiae* to *S. zoogloeoides*, 84% to 86%; *S. zoogloeoides* to K. pneumoniae, 87%. Protein name abbreviations: Gdpd, putative glycerophosphoryl diester phosphodiesterase; Pd, phosphodiesterase; Nsps, norspermidine sensor; RNAtr, putative RNA-binding transcriptional regulator; Tr, ArsR family transcriptional regulator; Gly, glyoxalase/bleomycin resistance protein/dihydroxybiphenyl dioxygenase; Tp, l,d-transpeptidase catalytic domain protein; RecO, DNA repair protein RecO; Padp, phenylactetic acid degradation protein; DUF389, DUF389-containing protein; Pmp, predicted membrane protein; Era, GTPase Era; Phd, putative HD superfamily hydrolase. Nucleotide sequence accessions top to bottom: MN628598, SHMI01000003.1, SLVX01000002.1, WUMK01000006.1, WUML01000002.1, and KP689347.

All bacterial genomes and plasmids (*n* = 610,187, downloaded March 2020) available in GenBank were searched for the *bla*_GPC-1_ and *bla*_BKC-1_-like genes, using DIAMOND v0.9.24.125 at a 70% identity cutoff (5). The *bla*_GPC-1/BKC-1_-like genes were identified in 19 assemblies and plasmids. In addition to the presence of *bla*_BKC-1_ in the originally reported plasmid from K. pneumoniae, the most similar sequences were found in two Shinella zoogloeoides chromosomes (81.7% and 85.4% amino acid [aa] identity, but if the duplication of part of the gene sequence is considered, the identity is up to 90.2%; see Discussion). The *bla*_GPC-1_-like genes were found in 14 different *Shinella* spp. genomes (S. granuli, S. kummerowiae, S. curvata, *Shinella* spp.; 80.1 to 93.3% aa identity), and two genomes whose global average nucleotide identity (gANI) analysis showed they are likely to be related to *Shinella* species and may have been misnamed (*Sinorhizobium* sp. RAC02 [84.9% aa identity] and uncharacterized *Rhizobiaceae* bacterium UBA3138 [78.9% aa identity]). Analysis of the two genes’ genetic environments in K. pneumoniae and P. aeruginosa showed the previously demonstrated association with both insertion sequences and plasmid-specific genes (Fig. 1). On the contrary, no insertion sequence or other genes indicating mobility could be associated with the homologues of *bla*_GPC-1/BKC-1_ found in *Shinella* spp. The *bla*_GPC-1_ and the *bla*_BKC-1_ homologs found in different *Shinella* spp. were located on the same chromosomal locus as indicated by strong synteny (Fig. 1). Sequence dissimilarities of 7% to ≥20% across this locus between species indicate a long-standing association with the *Shinella* genus (Fig. 1). Moreover, analysis of the sequences immediately up- and downstream of *bla*_GPC-1_ and *bla*_BKC-1_ showed high homologies with the corresponding loci in *Shinella*. The upstream and downstream regions of the *bla*_GPC-1_ gene shared 85% (44/52) and 86% (48/56) nucleotide identity with *S. granuli* DD12, while for the *bla*_BKC-1_ gene, the corresponding identities to *S. zoogloeoides* DSM287 were 80% (12/15) and 87% (87/100), respectively.

The GC content of the *bla*_BKC/GPC_-like genes from all *Shinella* isolates and their mobile counterparts ranged from 65.3% to 69.7%. This overlaps with that of the larger (±10,000 bp) genetic contexts in *Shinella* (63.8% to 66.6%) but not with that of clinical species carrying *bla*_BKC/GPC_ genes (59.3% to 60.5%).

Altogether, this indicates that the two resistance genes share an ancestor gene that have evolved separately into a more *bla*_BKC-1_-like gene in *S. zoogloeoides* and a more *bla*_GPC-1_-like gene in, e.g., *S. granuli*. It is therefore highly plausible that that *bla*_GPC-1_ and *bla*_BKC-1_ were mobilized from different *Shinella* spp.

**FIG 2:**
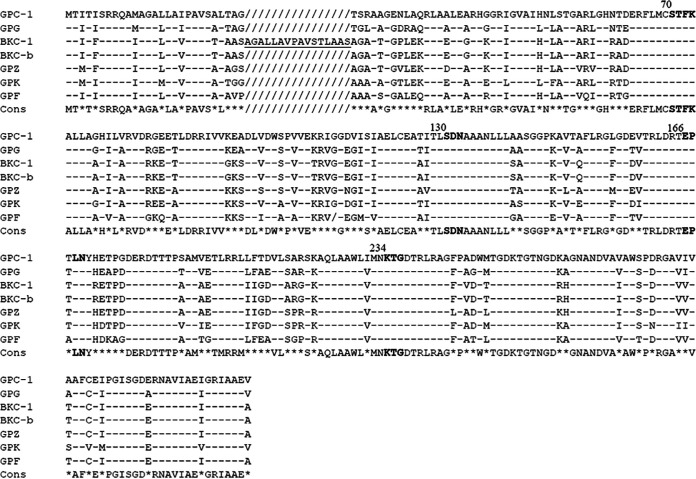
Amino acid sequence comparison between the different BKC/GPC-like enzymes. A dash represents an amino acid that is common among all variants, a slash represents a gap. The underlined sequence represents the duplication of the peptide Ala^12^ to Ser^27^ in BKC-1. Bolded sequences implicated the conserved motifs present in class A β-lactamases: ^70^STFK, ^130^SDN, ^234^KTG involved in the catalytic activity and ^166^EPxLN, involved in the Ω-loop (ABL numbering).

To evaluate the phenotype provided by the *bla*_BKC/GPC_-like gene and to find other variants and potential closer homologs, four different *Shinella* species were recovered from the public bacterial collection bank of the Culture Collection of the University of Gothenburg (CCUG), being *S. granuli* 56487, *S. kummerowiae* 56777, *S. zoogloeoides* 35204, and S. fusca 55808 (6–8). PCR experiments using degenerate primers were performed, and amplified β-lactamase genes (named *bla*_GPG_, *bla*_GPK_, *bla*_GPZ_, and *bla*_GPF_, respectively) were cloned into E. coli TOP10 using the pCR2-TOPO cloning kit (Thermo Fisher) and tested for the resistance phenotype using broth microdilution. A CarbaNP test (9) confirmed the ability of the GPC/BKC-like expressing clones to hydrolyze imipenem. All clones displayed a resistance profile against β-lactams, including amino- and ureidopenicillins, first- and second-generation cephalosporins and a low level of resistance against third- and fourth-generation cephalosporins, monobactam, and carbapenems. All clones remained susceptible to the cephamycin cefoxitin. In addition, the use of clavulanic acid or avibactam restored a complete susceptibility against amoxicillin or ceftazidime, respectively, a characteristic shared by class A β-lactamases (Table 1). This phenotype is in accordance with those reported for BKC-1 and GPC-1 (3, 4). Protein alignments using SeaView Software (Prabi, Doua, France) showed that GPC-1 was most closely related to the GPC-like proteins from *Shinella* sp. strian DD12 (93.3%) and *S. granuli* (92.6%), whereas BKC-1 shared the highest amino acid identity with *S. zoogloeoides* (85.4%) (see Fig. S1 in the supplemental material). All variants possessed the typical conserved serine/threonine kinase motifs and the motif involved in the Ω-loop formation of class A β-lactamases (10) (Fig. 2). Deeper alignment analysis showed that BKC-1 displayed a duplication of 16 amino acids, being the repetition of the protein segment from Ala^12^ to Ser^27^. Therefore, a putative ancestral protein was designed *in silico* and named BKC-b (Fig. 2). Aligning the BKC-b sequence with GPZ increased the amino acid identity up to 90.2% compared to 85.4% without the duplication (Fig. S1). Production of BKC-b in E. coli TOP10 showed a weaker resistance profile while having an increased activity against cefoxitin. The use of the I-TASSER (11) *in silico* tool predicted the tridimensional structures of both BKC-b and BKC-1 and showed that the duplication of the protein segment in BKC-1 modified the ligand binding site of the enzyme and probably led to the increased spectrum of activity observed in BKC-1 compared to that in BKC-b but the loss of its activity against cephamycins.

**TABLE 1:** MICs of the clones expressing the different BKC and GPC variants

Clone or strain	MIC (μg/ml)^*a*^													
	AMX	AMC	PIP	CEF	FOX	CXM	CTX	CAZ	CZA	FEP	ATM	IPM	MEM	ERT
E. coli TOP10	4	4	2	4	4	4	0.125	0.25	0.25	<0.125	0.125	0.125	<0.125	<0.125
pGPC-1	>256	6	64	>256	6	256	32	1	0.25	2	4	1	0.25	<0.125
pBKC-1	>256	4	32	>256	6	>256	32	4	0.25	1	2	1	0.125	0.125
pBKC-b	32	6	4	32	64	64	2	0.5	0.25	0.125	0.5	0.25	<0.125	<0.125
pGPG	>256	4	8	16	8	32	1	0.5	0.25	0.25	1	0.5	<0.125	<0.125
pGPK	>256	8	>256	>256	8	>256	32	0.5	0.25	0.75	8	1	<0.125	<0.125
pGPZ	>256	4	>256	>256	8	>256	32	1	0.25	0.25	16	2	0.25	0.25
pGPF	>256	6	128	>256	8	128	4	0.5	0.25	0.5	2	1	<0.125	<0.125

a AMX, amoxicillin; AMC, amoxicillin-clavulanic acid; PIP, piperacillin; CEF, cephalothin; FOX, cefoxitin; CXM, cefuroxime; CTX, cefotaxime; CAZ, ceftazidime; CZA, ceftazidime-avibactam; FEP, cefepime; ATM, aztreonam; IPM, imipenem; MEM, meropenem; ERT, ertapenem.

Here, we provide evidence that the genes *bla*_GPC-1_ and *bla*_BKC-1_ were most likely mobilized from members of bacterial genus *Shinella* into clinical species. This conclusion is based on the presence of a conserved locus containing a *bla*_GPC/BKC_-like gene in all investigated *Shinella* species, the lack of associated mobile genetic elements, and high amino acid and nucleotide identities to the clinical counterparts, but not so high that we could assign with confidence the exact origin species. However, it is highly plausible that the origins of *bla*_GPC-1_ and *bla*_BKC-1_ are *Shinella* species closely related to *S. granuli* and *S. zoogloeoides*, respectively. The resistance phenotype provided by the *bla*_GPC/BKC_-like genes is in line with mobilization and transfer driven by antibiotic exposure. The *Shinella* genus includes mesophilic, aerobic Gram-negative species mainly recovered from environmental samples. For instance, the studied *S. granuli* and *S. zoogloeoides* isolates were recovered from sludge in China, while the *S. kummerowiae* and *S. fusca* isolates were recovered from root nodules and domestic compost in Korea and Portugal, respectively (6–8). The presence of a natural and functional β-lactamase gene in this genus could be explained by the presence of β-lactam-producing microorganisms sharing the same niche (12). Additionally, we show that the BKC-1 protein presented a duplication of its Ala^12^-Ser^27^ segment, likely from a putative ancestral protein BKC-b. Hence, the *bla*_BKC-1_ gene may have evolved from *bla*_BKC-b_, likely under a selective pressure from β-lactams, eventually resulting in a more efficient enzyme. This mutation led, on the other hand, to the reduction of its activity against cefoxitin.

Emergence of new resistance genes, especially genes providing resistance to antibiotics of last resort, such as carbapenems, represents a major clinical threat. After initial emergence, they are likely to remain undetected and spread silently in the human microbiota for some time. When detected, they are often already widespread (13). Understanding the origin and mobilization history of as many and diverse clinically important resistance genes as possible could enable us to manage risks for future emergence events in a better way. The data presented here provides one additional piece in this large puzzle.

### Data availability.

The nucleotide sequence of the carbapenemases genes *bla*_GPG_, *bla*_GPK_, *bla*_GPZ_, *bla*_GPF_, and *bla*_BKC-b_ were submitted to GenBank with the following accession numbers, respectively: MT661611, MT661612, MT661613, MT661614, and MT661610.

## Supplementary Material

Supplemental file 1
