# Supplementary material for: The Class A Carbapenemases BKC-1 and GPC-1 Both Originate from the Bacterial Genus Shinella
Source: Antimicrob Agents Chemother. 2020 Nov 17;64(12):e01263-20. doi: 10.1128/AAC.01263-20 (PMC7674036; doi:10.1128/AAC.01263-20)
Supplement: Supplemental file 1 [file AAC.01263-20-s0001.pdf]

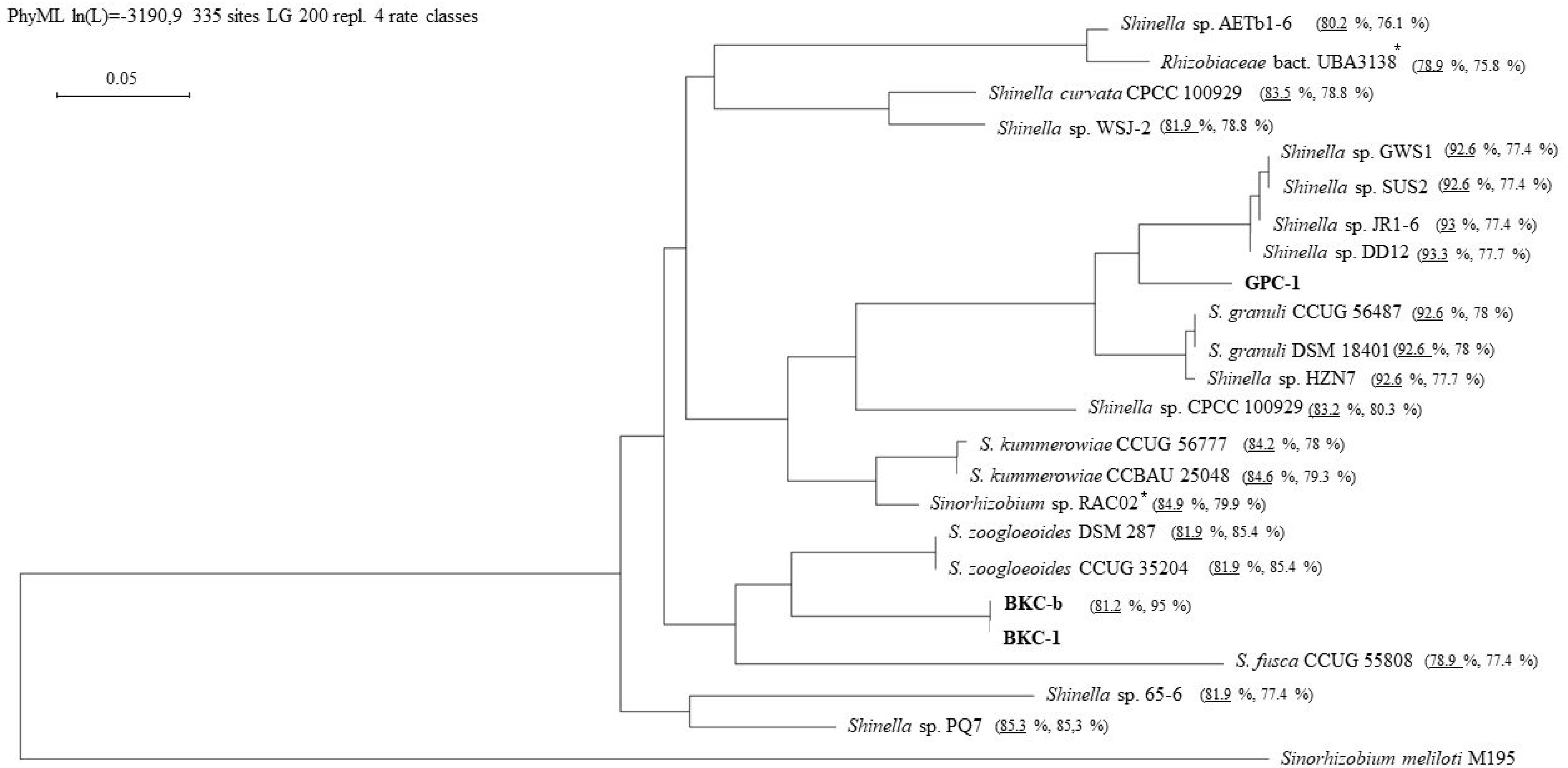

Figure S1. Phylogenetic tree obtained for different GPC/BKC-like enzymes including the putative ancestral protein BKC-b by maximum likely-hood method using the LG model (SeaView version 4 software) (1). Branch lengths are drawn to scale and are proportional to the number of amino acids substitutions with 500 bootstrap replications. The distance along the vertical axis has no significance. Percentage represent the amino acid identities shared by the enzyme with GPC-1 (underlined) and BKC-1. Accession numbers of the different isolates tested: BKC-1 (NG_048710.1), GPC-1 (MN628598.1), *Shinella* sp. DD12 (GCA_000496935.2), *Rhizobiaceae* bacterium UBA3138 (GCA_002364745.1), *Shinella* sp. SUS2(GCA_001262505.1), *Shinella* sp. GWS1 (GCA_001267455.1), *Shinella* sp. 65-6 (GCA_001898065.1), *Shinella* sp. MEC089 (GCA_004514425.1), *Shinella* sp. MEC087 (GCA_004514435.1), *Shinella* sp. HZN7 (GCF_001652565.1), *Sinorhizobium* sp. RAC02 (GCF_001713395.1), *S. zoogloeoides* PQ7 (GCF_003574625.1), *Shinella* sp. WSJ-2 (GCF_003432075.1), *Shinella* sp. JR1-6 (GCA_004284735.1), *S. granuli* DSM 184 (GCA_004341885.1), *Shinella* sp. CPCC 100929 (GCA_010994755.1), *S.* curvata (GCA_010994455.1), *S. kummerowiae* CCBAU (GCA_009827055.1), *Shinella zoogloeoides* DSM287 (GCA_009826855.1), *Shinella* sp. AETb1-6 (GCA_009826875.1). Sequences from *S. fusca* CCUG 55808, *S. granuli* CCUG 56487, *S. kummerowiae* CCUG 56777, *S. zoogloeoides* CCUG 35204 and BKC-b were deposited on Genbank (MT 661610-14). The tree was rooted with the class A β-lactamase from *Sinorhizobium melitoli* M195 (Genbank: CP004138.1). *gANI analysis suggests these isolates are mis-identified *Shinella* species

Reference-supplementary data

1 Gouy M, Guindon S, Gascuel O. 2010. SeaView version 4: a multiplatform graphical user interface for sequence alignment and phylogenetic tree building. Mol Biol Evol 27:221-224. doi: 10.1093/molbev/msp259.
